# Reply to Macé *et al*.

**DOI:** 10.1093/icvts/ivaf058

**Published:** 2025-03-13

**Authors:** Joeri Van Puyvelde, Filip Rega, Bart Meyns

**Affiliations:** Department of Cardiac Surgery, University Hospitals Leuven, Leuven, Belgium; Department of Cardiovascular Sciences, KU Leuven, Leuven, Belgium; Department of Cardiac Surgery, University Hospitals Leuven, Leuven, Belgium; Department of Cardiovascular Sciences, KU Leuven, Leuven, Belgium; Department of Cardiac Surgery, University Hospitals Leuven, Leuven, Belgium; Department of Cardiovascular Sciences, KU Leuven, Leuven, Belgium

**Keywords:** Fontan, Fontan failure, Ventriculo-arterial coupling, restrictive pathophysiology, systolic dysfunction, physiology

Dear Editor,

We appreciate the insightful letter by Macé and Lenoir [1] regarding our recent article on Fontan circulatory failure [2]. Their perspective, grounded in fundamental cardiovascular physiology principles, offers a valuable framework for understanding the complex pathophysiology of Fontan circulation.

The authors highlight two key concepts: ventriculo-arterial coupling and venous return resistance. These principles, indeed, provide a compelling explanation for the dichotomous observations in our study regarding right and left ventricular dominant morphologies.

The concept of contractility-afterload mismatch, as described by Sunagawa *et al.* [3], elegantly explains the progressive systolic dysfunction observed in right ventricular dominant patients. This mismatch, exacerbated in the Fontan circulation due to increased impedance, particularly affects the systemic right ventricle, corroborating our findings.

Regarding venous return, Guyton’s work on the profound impact of venous resistance is particularly relevant in the Fontan setting, as previously demonstrated by Macé *et al*. [4]. The authors’ assertion that chronic increase in venous return resistance leads to sustained unloading of the single ventricle and the development of a restrictive pathophysiology with diastolic dysfunction, regardless of its morphology, is intriguing and thought-provoking.

The crucial question raised is whether systolic dysfunction could potentially mask diastolic dysfunction in right ventricular dominance. Our experience aligns with this possibility but reveals a more nuanced picture. Patients with systolic dysfunction and failing Fontan circulation often develop diastolic dysfunction as well. However, we observe that these patients typically present with markedly dilated ventricles. Therefore, this diastolic dysfunction appears to be a consequence of systolic dysfunction and subsequent ventricular remodelling, rather than a primary result of chronic low preload. Conversely, the mechanism of diastolic dysfunction in left ventricular dominance seems distinct. These patients are characterized by a small, restrictive ventricle, as we previously described [5]. This stark contrast in ventricular morphology and function between right and left ventricular dominant patients suggests two different pathways leading to diastolic dysfunction.

In right ventricular dominant patients, the progression seems to be:

1. Systolic dysfunction due to contractility-afterload mismatch
2. Ventricular dilation and remodelling
3. Secondary diastolic dysfunction

For left ventricular dominant patients, the pathway appears to be:

1. Chronic preload deprivation
2. Development of a restrictive pathophysiology
3. Primary diastolic dysfunction

This difference in ventricular response to chronic preload deprivation between right and left ventricles is an area ripe for further research. It may be explained by their distinct anatomical and geometric properties, as well as potential differences in their adaptive mechanisms to chronic stress.

In conclusion, Macé and Lenoir’s letter provides a valuable physiological perspective on Fontan circulation failure modes. Their insights, combined with our clinical observations, underscore the need for a nuanced understanding of ventricular dysfunction in Fontan patients. We must consider not only systolic and diastolic components but also their potential interplay and the distinct pathways they may follow based on ventricular morphology. This comprehensive approach will be crucial for developing targeted therapeutic strategies and improving long-term outcomes in this complex patient population.

## Data Availability

There are no data in this letter.
